# Investigating Youth Sport Coach Perspectives of an Asthma Education Module

**DOI:** 10.1155/2018/2512010

**Published:** 2018-06-03

**Authors:** Francesca S. Cardwell, Susan J. Elliott

**Affiliations:** Department of Geography and Environmental Management, University of Waterloo, Canada N2L 3G1

## Abstract

Physical activity can reduce symptoms and improve wellbeing in people who have asthma, and organized sport is one way for children and youth with asthma to engage in exercise. While asthmatic youth may experience a number of barriers to sport participation, healthy physical and social sport environments supported by coaches can help asthmatic youth athletes maintain long-term engagement in activity. This paper reports results of an assessment of an online coach education tool related to air quality, physical activity, and allergic disease (e.g., asthma). Focus groups with youth team sport coaches in southern Ontario (*n* = 12 participants) were conducted to explore how users experience the module and short- and medium-term outcomes of implementation. Although coaches perceive the module as relevant, it is considered less valuable in certain contexts (e.g., indoor environments) or when compared with other coach education (e.g., tactical). Although broad asthma management behaviours (e.g., athlete medical forms) were recognized, specific module-identified prevention and management techniques (e.g., the Air Quality Health Index) were less frequently described. Ensuring environment and health coach education emphasizes athlete performance while reducing risk is critical to promoting module application and providing safe and enjoyable youth team sport spaces.

## 1. Introduction

Asthma is one of the most common chronic diseases of childhood worldwide [1, 2] and affects approximately 600,000 Canadians under age 12 [3]. In Ontario, one of Canada's largest provinces with a population of 14 million [4], asthma prevalence increased by 70.5% between 1996 and 2005, attributable, in part, to an increase in children's incidence [5]. It is projected that almost 1 in 8 Ontarians will have asthma by 2022 [6], leading to calls for public health strategies for asthma prevention and management [5]. A growing body of evidence indicates that environmental changes, such as temperature change, extreme weather events, and air pollution, are likely to impact respiratory health [7–11], particularly in vulnerable groups such as children [7, 12]. Impacts will vary geographically; for example, urbanization and vehicle emissions in high-traffic areas are correlated with an increased frequency of respiratory allergy [13, 14].

Physical inactivity is also associated with asthma symptoms in asthmatic children and youth [15–17]; although physical activity can act as a trigger for those whose asthma is not well managed, there have been calls for a “prescription” for exercise [15], as physical activity is an important component of a comprehensive asthma management program and can not only improve asthma management and wellbeing of asthmatic patients, but also minimize other chronic health risks associated with sedentary behaviours [18, 19]. Children and youth often choose to participate in organized sport, and there is considerable evidence demonstrating the physical and social benefits of participation [20, 21]. In North America, children's organized sport outside of school is largely run through community-based programs and youth sport organizations [21], led primarily by volunteer coaches and athlete parents, although (in fewer, and more competitive instances) subsidized coaches exist.

While children and adolescents with asthma may experience an increased number of barriers to physical activity and sport participation compared with nonasthmatics (e.g., hesitation to participate in highly competitive environments) [19, 22], well-managed asthma in a supportive environment created by coaches, teammates, and sport providers can help asthmatic youth athletes perform in, enjoy, and maintain physical activity into adulthood [23].

In order to address the growing public health concerns associated with the links between physical activity, respiratory disease, and the physical environment (e.g., climate change), the organization Clean Air Champions, in partnership with the Coaching Association of Canada, developed the world's first online coach training tool focusing on air quality and respiratory disease management in sport. The Air Aware Coach Module was developed and peer-reviewed by Canadian coaching, air quality, and medical and healthcare experts to increase awareness of air quality and allergic disease associated with sport participation [24, 25]. (As of the date of submission, the module is not currently offered by the Coaching Association of Canada as the Clean Air Champions organization has been terminated due to lack of funding. At the time of research, the module was live online.) The module provides tools, resources, and training on how to reduce asthma attacks, support optimal performance, and ensure healthy environments for athletes. Coaches are encouraged to apply content in their seasonal plans [24]. It was released in 2013 and is completed online via the Coaching Association of Canada website, and participants receive an Air Aware certification upon completion. The module is available across Canada for $15 (CAD) and is offered in both official languages (French and English).

This module is the first online coach education related to air quality, physical activity, and allergic disease. Investigating how coaches understand the content, value and prioritize environment and health risks in sport (specifically, asthma), and apply module recommendations with respect to asthma management and prevention is therefore critical. In order to assess the impact of the module with respect to coach knowledge, attitudes and practices related to asthma, and physical activity, the objectives of this paper are (1) to understand the short- and medium-term outcomes of the Air Aware Coach Module and (2) to investigate the user experience of the module's implementation.

## 2. Materials and Methods

This research investigates responses from focus groups and one semi-structured in-depth interview with recreational and competitive youth team sport coaches. Focus groups were conducted in March 2015 in the Greater Golden Horseshoe Region of southern Ontario. While the Air Aware Coach Module is available for coaches across Canada [25], participants were recruited from the Greater Golden Horseshoe region due to the quantity and variety of youth team sport organizations, and as these communities demonstrate diversity in their sociodemographic profile and physical environment. Recruitment was conducted to ensure maximum variation in sampling.

Participants include community-level recreational and competitive coaches (*n* = 12) of organized youth indoor and outdoor team sport. Purposive sampling was employed, and participants were recruited through sports organizations in the sample region. Inclusion of clubs in the sample frame was restricted to those providing recreational or competitive, outdoor or indoor, organized child and youth team sport. After compiling a list of organizations (*N* = 219), a random sample of clubs was contacted by email using a random number generator and asked to distribute an advertisement to coaches. Coaches whose email addresses were provided on organization websites were contacted with an information letter by email. Snowball sampling was used to complete the sample and fill gaps in participant characteristics.

Coach participants completed the Air Aware Coach Module and were invited to participate in a follow-up in-person (*n* = 6 participants) or online focus group (*n* = 5 participants) (depending on geographic location and personal availability). The focus groups were held at least three months following the provision of the module so that coaches would have an opportunity to apply content in their training. The cost of the module was subsidized for all participants. The in-person focus group was conducted in a public meeting room at a local library, while the online focus group used the online meeting website https://www.gotomeeting.com. One coach was unavailable for the focus groups, so he/she participated in a semi-structured interview (using the same interview schedule). Focus groups began with general conversation related to participant demographics and sport participation in order to increase rapport and comfort between participants and the researcher. The focus groups and interview were digitally recorded, transcribed verbatim, proofed, and coded using Nvivo for Mac. Prior to coding and thematic analysis, the coding manual and major themes that emerged in the focus groups and interview were discussed with another member of the research team in order to reduce possible bias. This research was granted ethics clearance from the University of Waterloo Research Ethics Committee, and the research was conducted with participants' understanding and consent.

Short- and medium-term outcomes and the user experience of the module were investigated. Short-term outcomes related to coach knowledge and attitudes toward the module, air quality, respiratory health, and sport were explored. Few specific probing questions were asked, and the discussion was open-ended and flowed naturally. To investigate medium-term outcomes, participants were provided with asthma in sport scenarios that described module content (Boxes 1 and 2) and were asked to discuss asthma and athlete management in these scenarios. More specifically, we wanted to understand what information from the module, if any, was recalled, and how it may have impacted attitudes or encouraged specific behaviours related to asthma in sport. There were 2 scenarios, broken down into parts A and B. For each scenario, part A was read to participants, and the group was provided with a hard copy. After discussion related to part A, part B was read and discussion followed. Finally, user experience was explored to understand perceptions of module implementation, barriers related to content application, and coach recommendations for future module development.

## 3. Results

Findings are presented in four sections. An overview of participant characteristics is followed by short-term outcomes of the Air Aware Coach Module. Medium-term outcomes are then reported, followed by themes related to user experience. Results are presented using participant quotes to illustrate themes that emerged both deductively and inductively from the focus group transcripts.

### 3.1. Participant Characteristics

Overall, *n* = 12 coaches participated in the online (*n* = 5) or in-person focus group (*n* = 6), or the in-person interview (*n* = 1) (Table 1). Three coaches worked with a single team in a head coach role, while four served as assistant coaches. Five coaches worked with multiple teams, in both a lead and assistant capacity. No participants worked solely in a recreational program, although four were involved with both competitive and recreational programs, and often in multiple sports. The majority of coaches (*n* = 7) were involved with soccer as their primary sport, and the coaches were involved with children from a range of age groups between 8 and 18 years.

### 3.2. Short-Term Outcomes

Focus groups continued with a broad discussion of participants' perceptions of the relevance of the module to their coaching experiences and activities. Coaches generally believed elements of the module to be valuable. One coach suggested the module “was relevant given that the sport [they coached] is played outdoors, [and] air quality is an important factor in that it can affect performance” (Soccer Coach). Coaches often spoke to their experiences either as athletes, or with athletes with asthma they have coached. Social factors, such as the possible stigmatization of asthmatic athletes, were identified when emphasizing the module's value:

*More while I was a player, I had asthma and there were times it felt like you couldn't say you have asthma… you would hide your puffer… and it wasn't as accepted to ask the coach for a 10-minute break… and so I think the more it's understood that it's a real thing that certain kids struggle with it, the more appropriately we can handle it… I remember not really wanting coaches to know, or teammates… Boys especially will pick on anything you can pick on in a group setting like that, so I think if you're the one kid with asthma or you're caught wheezing there is kind of a stigma attached to that, especially with being in a vulnerable situation around other teenage boys, and not all parents are there. It's easily picked up on by boys that make fun of each other.* (Baseball Coach)

The need for increased education and awareness of the risks associated with asthma was also identified:

*It's a good module for other coaches to experience. A lot of people don't know too much about asthma and as we see asthma growing… I think it's really important for coaches to actually get an education about what asthma is and how can it be treated.* (Soccer Coach)

While participants generally perceived the module to be valuable, some (*n* = 3) declared its relevance was limited to certain contexts. This attitude was closely tied to coaches whose sports were predominantly played indoors (e.g., basketball) and less reliant on cardiovascular endurance (e.g., curling). While they identified that the module “did talk a little bit about indoor sports” (Curling Coach), it was not perceived as relevant for their context:

*It probably wasn't as strongly relevant to my sport, firstly being that it's an indoor sport and that you don't have the same kind of issues… you know the airborne elements, obviously they would still exist within an indoor space, but probably not to the same degree. As well, we don't have to deal with the same things as smog and things like that. So you do have issues but probably not as strongly related to any outdoor sport.* (Curling Coach)

Although many asthma-related themes from the module are relevant in indoor and outdoor contexts, and a component of the module specifically focused on indoor environments and associated air pollutants, indoor coaches generally articulated that the indoor environment was not sufficiently covered in the module. For example, this coach of a gym-based sport described insufficient coverage of asthma in their sport:

Researcher: Would you have found it more useful if it had information related to triggers in a gym environment, for example?

*Participant: Absolutely, and I think even if you're looking at other sports too, it would be beneficial because you're not always training outside… Like in wintertime if you're an outdoor sport and you're playing indoors.* (Basketball Coach)

Further, although climate change is discussed in the module with respect to respiratory health, athlete vulnerability, and increasing temperatures and air quality (e.g., particulate matter, ground-level ozone), coaches did not mention climate change when asked about the module's relevance. Similarly, although specific preventative and management behaviours such as using resources like the Air Quality Health Index (AQHI)—a community-level health protection scale designed to provide air quality and health messages (see: http://www.ec.gc.ca/cas-aqhi/default.asp?lang=En&n=065be995-1) [26]—were a significant theme of the module, coaches did not identify asthma management behaviours until probed.

### 3.3. Medium-Term Outcomes

To investigate medium-term outcomes, asthma in sport scenarios was discussed by participants. Part A of scenario 1 (Box 1) describes a 14-year-old male hockey player who has not been using his reliever medication (e.g., an inhaler) and has seen a reduction in his performance. The player is complaining about fatigue and is sitting out of games implying he is not fit to compete, but the coach observes no common signs of asthma. When asked to speculate why this player is not using his reliever medication, participants articulated that the player might not attribute the symptoms to asthma or feel a need for medication. A soccer coach described that they “wouldn't have even thought the asthma thing right away,” and they would have considered other factors such as “what's going on at home… healthy eating right before practice… mental grit, what's going on if [they're] fatigued… and then maybe eventually his health”.

Coaches also described potential stigmatization related to asthma. A soccer coach identified that “maybe he wants to hide it from his teammates,” and others discussed the possible fear of being stigmatized, perceived as weak, or having reduced playing time during games:

*So I think what really comes in is you see some of those competing pressures that this individual has in terms of the fact that maybe there is peer pressure within their circumstance on the competitive team but as well there is also the fact that you have him being a high-ranked athlete with the scouts being present and things like that. So it might be a sign of weakness or a sign of impeding their performance in some ways, it might be an attitude that the athlete has within their mind.* (Curling Coach)

Other reasons described include medication cost, frustration related to medication need, and previous ineffective medication use impacting perceived necessity:

Participant: Sometimes teenagers at this age, if it runs out they may not tell their parents.

Researcher: Why do you think that would be?

Participant: Cause they don't want to take it anymore… or it's a pain in the butt, and depending on his family situation, like at school we see it all the time with EpiPens. Oh, we just haven't gotten to the doctor, and like seriously this is your EpiPen! This is your lifeline! Oh yeah, we're just so busy we can't get there…

Researcher: Like perceived as lower on the priority list?

*Participant: Especially if he hasn't had major attacks.* (Soccer Coach)

Part B (Box 1) describes this player as the only player diagnosed with asthma using reliever medication. At a tournament the coach overhears discussion amongst teammates indicating social stigma around the player's medication use. While coaches identified possible stigmatization during part A, they were also asked about their role in managing the situation. The module emphasized coach support for asthmatic athletes, and participants echoed the value of inclusive social environments that ensure player comfort during medication use:

*I would make sure that it's not tolerated on the team because there could be many other reasons, not just his inhaler. There could be another kid who can't afford the top line hockey equipment and that shouldn't be an issue either.* (Basketball Coach)

Further, the importance of communication with the asthmatic player and possibly teammates and parents was described:

*I think this kid that's saying it, you might have to have a little one-on-one conversation with him and make sure that they understand what asthma is and what it's all about and the risks and the dangers, cause he may not realize what it is. Like 14-, 15-year-old kids if they've never had any experience with it, they're pretty, for lack of a better word, they're ignorant about it… He may not fully understand it.* (Soccer Coach)

Scenario 2 (Box 2) discusses a hot day early in an 8 to 10-year-old girls' soccer season. A new player is attending and her mother describes her symptoms before leaving practice. Participants were asked about important factors to consider in the scenario. Unsurprisingly given the module content, heat and air quality were mentioned by coaches. For example, a basketball coach described “keep[ing] things light because it's a hot and muggy day,” indicating consideration of the physical environment when session planning. Despite identification, many coaches did not focus primarily on the physical environment and questioned the parent's absence, especially given the age of the children:

*First of all, the mom should not be leaving the practice, especially at the age of 8 to 10, and even more so because of the hay fever. I think the parents should feel a bit more liable and show a bit more accountability and stay at that session.* (Soccer Coach)

When coaches were asked more specifically about behaviours to help the player, multiple possible actions were described. The need for a completed medical form and communication with the mother were expressed:

*Although the mother is in a rush, still emphasizing the importance of having the dialogue and getting as much information as possible, and then depending on the protocol of your club or team, getting some kind of paperwork health document filled out as soon as possible.* (Curling Coach)

While some coaches identified allowing the child to participate in training, others were less comfortable without completed medical documentation. This discussion highlighted the tension between ensuring athlete development, managing health risks, and organization guidelines/policy.

When asked more specifically if their organization affiliations enforce medical form completion, coaches described that while policy may exist, often the deadlines are ignored, coaches do not comply, and compliance is not enforced at the organizational level:

Participant: Sometimes at organized clubs they are kind of like, yes we get kid's health forms, but they come late. At school kids aren't even allowed to tryout for the team unless you have a health form. So that kid wouldn't be allowed to train if it was a school thing.

Researcher: But in an organization?

*Participant: Sometimes it just kind of flows into happening.* (Soccer Coach)

When asked if their club had medical form policy, participants were often unsure. Regardless, coaches described completing medical forms for their own records:

Researcher: So, there isn't policy around that?

Participant 1: Do you know [asking Participant 2]? I don't know.

Participant 2: Policy around what?

Participant 1: Of having health forms submitted before they train at the club?

Participant 2: Like I've always had one done. Not from the club, I did a personal thing that said any asthma, diabetes, Epipen, are they allergic to certain fruits?

Participant 1: For teams yes, but the way clubs are going with these divisional programs…

Participant 3: There's nothing making us do that.

*Participant 1: … sometimes you get like, oh here's a new kid and they show up for the first time and you haven't seen anything about it… and that's to me, from a liability standpoint, it's super dangerous (laughter). But it happens at the soccer-level all the time.* (In-Person Focus Group Discussion)

Part B (Box 2) stated that similar weather is forecast for the following Saturday, and the AQHI reads 7 the morning of practice. Four players have respiratory allergy and two have asthma, and the session is at noon. Participants were asked about the AQHI and possible coach behaviours. Some coaches described that “before this Air Aware Module [they] didn't even know what the AQHI was” (Curling Coach), and prior to part B of scenario 2, the AQHI was not identified by participants. When probed, coaches often provided a vague description or were not confident in their description:

*The ones with respiratory allergies or asthma are going to feel it more the higher the scale climbs, right? So personally, I don't know what a 7 would feel like… versus an 8, 9 or 10.* (Soccer Coach)

When asked about a reading of 7, others were able to identify broad themes related to the index but could not describe specific recommended behaviours:

*I see 7 out of 10 and so I know that's high… other than knowing where a 7 lies on the scale, I don't know what that feels like, I don't know how it affects the kids directly, just as I'm sure there are other impacts such as where the field is located, what kind of practice you are going to be running, all that kind of stuff.* (Baseball Coach)

Although participants identified the AQHI's value and that it was “another resource to check” (Basketball Coach), a lack of coach autonomy was identified as a barrier to its use. Coaches described that decisions are often made at the club level related to training cancellation and scheduling:

*I think going forward now knowing more about it I would be more likely to maybe make a note about it. I think in the past I wouldn't really be motivated to bother checking… it might be something that I would check to see if it was actually as bad as it felt out, and that if I should maybe alter my practice a little bit, but … I really don't know if I would ever check it otherwise, and even saying that it's usually someone else's decision if it's going to be closed that day, like a league or the City will send out a notice that games won't be allowed.* (Baseball Coach)

Coaches of indoor sports also described the AQHI as less relevant:

*Probably if I ever coached sports… involving an outdoor environment I would pay more attention to it.* (Curling Coach)

According to the AQHI, a reading of 7 suggests reducing/rescheduling strenuous outdoor activities for those at risk (e.g., children) or if symptoms (e.g., coughing, throat irritation) are experienced [26]. Based on this reading and the training conditions, the module recommends possible coach behaviours. Coaches echoed some of the module recommendations, including allowing players to take breaks and “more hydration times during the session” (Soccer Coach), modifying session plans to focus on skill development versus endurance training, communicating with parents and players with respect to athlete asthma support, and monitoring players for symptoms:

*Sometimes when it's been really hot and we have our training sessions, I have a “go-to really hot practice session,” where it's not as cardio-focused and… there are some sections of the field that are better shaded than others. You kind of move your stuff to certain areas… and you modify your session accordingly. It is quite dangerous to train at that time, cause 7 I think is kind of in the borderline danger zone? If I remember, I'm not going to say I remember 100%, but I feel like it's getting up there. But making it not as cardio fitness focused would be a good idea and to keep a very watchful eye on those players and have regular check-ins.* (Soccer Coach)

Some of the more specific terminology or guidance from the module was not described. For example, the use of an individualized Asthma Action Plan, or following the 1-2-3 rule (using a rescue inhaler once before and a second time during activity, but ceasing participation if needed three times), was not identified.

Possible structural changes were also identified. For example, coaches described changing the training venue location and time, or moving training indoors for improved air quality. Club policy and organizational control were identified as barriers to these behaviours, specifically related to facility availability and training time and location:

Participant: In an ideal world, you get to choose your facilities, you get to choose your time slots, you get to create a really positive inclusive environment.

Researcher: Now when you say in an ideal world, what are some of the barriers you see to doing those things?

Participant: What's the right word, logistics from a club. Trying to slot everybody in, and use for [the fields is] growing and that's a fantastic thing, but we had three or four age groups training at the same time with limited fields, especially in those odd seasons like Fall and Spring when you are not allowed on certain grass fields. It can get dicey with what's available.

Researcher: And do you think that's something other sports see as well?

*Participant: Other sports where you need permits for stuff, for sure.* (Soccer Coach)

Finally, social dynamics related to environment and health behaviours (e.g., cancelling training) were also identified. One coach explained perceived parent judgment:

*If I am being honest I think there is probably a little bit of a social effect as far as coaching and if you have, you know, parents that are more old-school… cancelling a practice for older boys because it's, you know, cause it's too smoggy or too hot out I think there is a little bit of, you could get a little bit of backlash or a little bit of murmur from the old-school parents, or parents that used to be coaches, where “we never cancelled practices back in our day”… not saying that they're right, I'm just saying that might be something that I can think of that would probably cross my mind if I was cancelling a practice because of the AQHI.* (Baseball Coach)

This attitude highlights the tension between managing athlete health and parental and player expectations and maximizing performance (and team success). The module described avoiding the “tough it out” attitude, in order to reduce stigma, and ensure players communicate symptoms with teammates or coaches. Ensuring well-managed social and physical environments is critical to both team success and reducing health risks in sport.

### 3.4. User Experience

Most coaches did not report problems related to module usability. While one participant described their preference for classroom learning, this theme was only identified once:

*I don't love learning online, I like in a classroom setting or having someone instruct you… it makes you interact with the material versus just skimming through and reading and answering multiple choice questions. It just gives you another way to kind of really absorb the information and let it hit home, so I would prefer if it was added on into a classroom setting.* (Basketball Coach)

When they were asked if they participated in the optional components or accessed additional resources, the uptake was fairly low; some coaches saved the resources for later reference, while others did not read the content. One coach recommended resource availability following module completion:

*I think it would be important… to be able to provide resources that coaches can refer back to… or they could disseminate, whether that being to athletes, parents or coaches just to broadcast at least the basic messages as widely as possible.* (Curling Coach)

Coaches were asked about barriers to module participation. While some articulated that $15 was reasonable compared with other coach education, others identified cost as a barrier as “if it was put out there for $15… your pool of people you'd be getting information to would be limited” (Soccer Coach). Coaches discussed the value of organization subsidization of coach education costs and identified that asthma coach education would not be prioritized if paying out-of-pocket.

Similarly, coaches described a lack of interest in asthma education compared with other possible courses, such as tactical or technical skill development:

*I don't know that as a head coach that would be something I would have sought out and purchased on my own, but I think more since it's covered for research or if it was covered by the club to go do, I don't think as just a head coach I would necessarily look into it and choose to spend my own money on that… If I was going to spend my own money or if I was going to try to prove a need to justify a regular course, I would be more comfortable and probably more prone to look into more strategy or drills-type of courses, more than even concussion or asthma or anything like that.* (Baseball Coach)

Participants generally recommended the module, particularly if subsidized. In order to improve the module or increase engagement, coaches identified combining asthma content with sport-specific coach education and emphasizing the benefits to specific sports and team performance. Coaches believed this would increase interest in Air Aware content and provide cobenefits related to coaching their sport. A summary of results is presented in Table 2.

## 4. Discussion

This exploratory work has not only increased our understanding of how environment and health risks (specifically allergic disease and asthma) are understood amongst coaches in Ontario but offers an applied example of how organized youth team sport coaches perceive and apply an environment and health online coaching module. This research qualitatively assessed the impact of the Air Aware Coach Module. The module aims to provide an overview of issues related to the environment, respiratory health, and physical activity, supply protocols and resources for coaches to prevent or reduce asthma attacks, and increase coach understanding of risk factors as part of their due diligence [24]. This research investigated how coaches understand, recall, and apply module content in their team and athlete management and their recommendations for future module development. Understanding how coaches use the module and manage child and youth asthmatic athletes is critical to maximize athlete performance and ensure healthy and safe sport environments for participants.

While other health-focused coach education exists (e.g., Making Head Way Concussion eLearning Series, offered in Canada by the Coaching Association of Canada), to our knowledge, the Air Aware Coach Module is the first and only coach education tool focusing on air quality and respiratory health [25]. This module is situated amongst other asthma education tools targeted toward a range of audiences. For example, Bruzzese et al. discuss an Asthma Self-Management for Adolescents program aimed to improve adolescent self-management skills [26], while Swerczek et al. describe a nurse telephone coaching intervention that supports asthma self-management behaviours for parents [2]. Education programs for asthmatic children, parents, and stakeholders in the school environment also exist [27–30]. The successful application of this module therefore fills a significant gap as it engages a population that may not have formal coach training [31], or asthma management or child and youth health education. How other organized team sport stakeholders (e.g., sport organization guideline and policy-makers) prioritize or understand asthmatic athletes is an avenue for future research.

When coaches were asked about module relevance, broad themes related to asthma management in sport (e.g., athlete stigmatization) were identified. Specific details (e.g., the AQHI, applying an Asthma Action Plan) were less frequently described, even when probed. Coaches often drew on their own asthma symptoms and behaviours or experience coaching asthmatic athletes (e.g., use of reliever medication). Given all participants were involved with competitive teams, they may have longer-term relationships with athletes and exhibit a deeper understanding of athlete health concerns compared with a recreational coach sample (who typically coach players for a single season). Understanding how coaches from recreational environments value and apply the module may provide a deeper understanding of how coaches engage with and understand athlete health, encourage buy-in from different coach populations, and ensure both competitive and recreational athletes experience healthy physical activity and sport participation. This is critical given the number of children involved in organized sport (approximately 51% of Canadian children) [32] and the unique barriers recreational coaches may face to pursuing coach education (e.g., time availability given the voluntary nature of recreational coaching).

The imbalance of competitive and recreational coaches may also have influenced focus group dynamics, as competitive coaches may be more likely to emphasize team performance and results compared with recreational coaches. This emphasis could also influence how coaches form relationships with and manage asthmatic athletes. Future research focusing specifically on recreational coach perceptions and experiences will allow for increased understanding of how asthmatic athlete management is prioritized in different sporting contexts in Ontario. Further, we know undesirable coaching behaviours, injury, lack of fun, and excessive pressure are possible reasons for dropout and decreased sport satisfaction for youth athletes [31, 33, 34]. While some competitive coaches may be familiar with their athletes' health and social behaviours, some may engage in risky management behaviours (e.g., encouraging athletes to compete through physical pain) or interact more frequently with athletes who are healthy or do not disclose their symptoms [23]. This was emphasized when coaches reported increased interest in education focusing on improved team performance compared with health in sport. This is unsurprising, as while social developmental outcomes beyond on-field success are important to coaches [35], athlete development and enhanced performance are the primary objectives in competitive sport environments [36].

When asked about future coach education, participants prioritized other health outcomes (e.g., concussions) or tactical or technical skill development in their particular sport. While the module includes sport-specific components, it is purposefully designed to include coaches of all sports (e.g., team/individual, indoor/outdoor). Certain content was not perceived as relevant, and coaches described possible increased engagement if content was specific to their interests or incorporated in preexisting sport-specific education. Given the value competitive coaches put on team success and player improvement in addition to social development [37], emphasizing the links between well-managed asthma and team success and improved individual performance should be a major component of future coach education.

Other research [19, 22, 38] has described the possible barriers (e.g., organizational policies, avoidance of symptoms) that children or youth with asthma or respiratory disease may face related to participation in and enjoyment of physical activity. While children with asthma are generally less active than nonasthmatic youth [19], evidence indicates that physical activity can act as a protective factor against asthma development [16], and medical professionals recommend well-managed participation in physical activity for both respiratory and other health benefits [15, 39, 40]. This gap between evidence and behaviours (perceived barriers to involvement versus long-term physical activity) highlights the need for coach education emphasizing well-managed asthma and the provision of safe physical and social environments.

This research focused heavily on the role of the coach in understanding and managing respiratory health risks in youth team sport. While coaches are fairly autonomous, focus group participants described the role of structural factors in their ability to manage asthmatic athletes (e.g., club policy for practice/game cancellation). Further, coaches identified the value of organization subsidization of the module's cost in order to increase coach participation. While this may be possible in certain contexts, clubs may not have available resources for coaches to complete supplemental education, particularly given the challenge of declining sports participation [41] and the competition for often scarce resources between increasing participation costs, coach education, facility maintenance, and competition entry fees [32].

Finally, while focus group discussions indicate coaches aim to improve their athletes' ability to execute their sport in a safe environment, a limitation of this research exists in the social dynamics associated with focus group participation. Coaches often described asthma management or health-promoting behaviours when discussing the scenarios presented; however research participants tend to provide socially desirable responses, especially when the scope of the work involves a sensitive topic [42]. While the scenario-based focus groups are a good indicator of coach attitudes and behaviours, participant intentions may not dictate exactly how they would behave in practice or with different contextual factors. In competitive environments where winning is heavily emphasized and valued, some coaches may still not manage asthma according to Air Aware guidelines, potentially increasing athlete vulnerability.

## 5. Conclusions

In conclusion, ensuring environment and health coach education emphasizes how coaches can work with athletes to maximize performance while reducing risk is critical to promote module content application in youth organized sport. Improving coach understanding and management of environment and health issues, specifically related to asthma management, will help reduce athlete vulnerability, provide safe and enjoyable youth team sport spaces, increase sport participation and performance, and ultimately reduce the public health burden associated with physical inactivity and respiratory disease in Canada.

## Figures and Tables

**Box 1:**
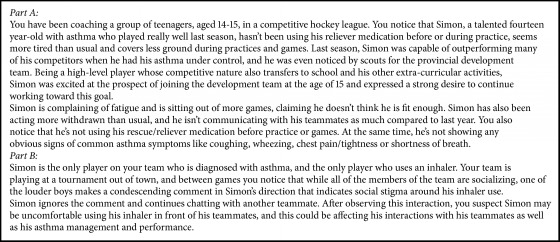
Focus group discussion scenarios—scenario 1, parts A and B.

**Box 2:**
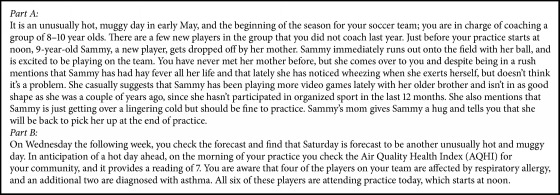
Focus group discussion scenarios—scenario 2, parts A and B.

**Table 1:** Participant demographic and coaching breakdown.

Participant Gender	Male	5
	Female	7

Focus Group Involvement	In Person	6
	Online	5
	Interview	1

Coach Position	Head	3
	Assistant	4
	Both	5

Level Coached	Recreational	0
	Competitive	8
	Both	4

Sport Coached	Soccer	7
	Basketball	1
	Baseball	1
	Curling	1
	Multiple Sports Coached	2

Age Group Coached	8 to 10	2
	11 to 13	1
	14+	5
	Multiple Age Groups Coached	4

**Table 2:** Summary of results.

Results section	Major themes explored	Subthemes explored
Participant Characteristics	Gender, Coach Position, Level Coached, Sport Coached, Age Group Coached	

Short-Term Outcomes	Module Relevance/Value	
	Personal Experiences	
	Social Factors	
	Increased Education/Awareness of Asthma	
	Limitations of Relevance	Context Specific, Key Components Not Identified (Climate Change, Asthma Management Behaviours)

Medium-Term Outcomes	Medication Use (Scenario 1)	Not Necessary for Symptoms, Stigmatization/Perceived as Weak, Medication Cost, Frustration, Ineffective Medication Use
	Coach Stigmatization Management Role (Scenario 1)	Inclusive Environment, Communication
	Team Management (Scenario 2)	Physical Environment (Heat, Air Quality), Role of Parent, Medication Information Form, Communication, Athlete Participation, Role of Organization (Policy, Non-Compliance)
	Asthmatic Player Management (Scenario 2)	Coach Behaviours (Increased Breaks, Hydration, Modified Training Content, Communication with Athletes and Parents), Resources (AQHI, Asthma Action Plan, 1-2-3 Rule), Barriers to Behaviours (Coach Autonomy, Content Relevance, Club Policy and Organizational Control), Facilitators to Behaviours (Structural Factors, Social Factors)

User Experience	Module Usability	
	Learning Preference	
	Additional Resource Use	
	Barriers to Participation	Cost, Interest in Content
	Recommendations	Long-Term Resource Accessibility, Combining with Sport-Specific Content, Benefits to Team Emphasized
